# Effects of a Multilingual Information Website Intervention on the Levels of Depression Literacy and Depression-Related Stigma in Greek-Born and Italian-Born Immigrants Living in Australia: A Randomized Controlled Trial

**DOI:** 10.2196/jmir.1527

**Published:** 2011-04-19

**Authors:** Litza A Kiropoulos, Kathleen M Griffiths, Grant Blashki

**Affiliations:** ^4^Nossal Institute for Global HealthThe University of MelbourneMelbourneAustralia; ^3^Depression & Anxiety Consumer Research Unit, ehub: emental health Research & DevelopmentCentre for Mental Health ResearchThe Australian National UniversityACTAustralia; ^2^School of Psychology & Psychiatry and School of Primary Health CareMonash UniversityMelbourneAustralia; ^1^The University of MelbournePsychological SciencesMelbourneAustralia

**Keywords:** Depression literacy, depression-related stigma, immigrants, Internet-based interventions, depression, randomized control trial

## Abstract

**Background:**

Little is known about the efficacy of Internet-based information interventions in increasing depression literacy or reducing depression stigma and depressive symptoms in people from non–English-speaking backgrounds.

**Objective:**

Our objective was to investigate the effects of Multicultural Information on Depression Online (MIDonline), an Internet-based multilingual depression-specific information resource, on depression literacy, depression stigma, and depressive symptoms in Greek-born and Italian-born immigrants to Australia.

**Method:**

In all, 202 Greek- and Italian-born immigrants aged 48 to 88 years were randomly allocated to an online depression information intervention (n =110) or a depression interview control group (n = 92). Participants allocated to the information intervention only had access to the website during the 1- to 1.5-hour intervention session. The primary outcome measures were depression literacy (depression knowledge), personal stigma (personal stigma toward people with a mental illness), perceived stigma (participants’ views about the probable attitude of the general community toward people with mental illness), and depressive symptoms. Depression literacy, personal and perceived stigma, and depressive symptoms were assessed at preassessment, postassessment, and at a 1-week follow-up assessment. The trial was undertaken at Monash University, Melbourne, Australia. Randomization and allocation to trial group were carried out using a computer-generated table.

**Results:**

For depression literacy, there was a significant difference between the MIDonline and the control group with those in the MIDonline intervention displaying higher depression literacy scores postassessment (F_1,178_ = 144.99, *P* < .001) and at the follow-up assessment (F_1,178_ = 129.13, *P* < .001) than those in the control group. In addition, those in the MIDonline intervention showed a significantly greater decrease in mean personal stigma scores postassessment (F_1,178_ = 38.75, *P* < .001) and at the follow-up assessment (F_1,176_ = 11.08, *P* = .001) than those in the control group. For perceived stigma, there was no significant difference between the MIDonline intervention and the control group at postassessment (F_1,178_ = 0.60, *P* = .44) and at the follow-up assessment (F_1,176_ = 1.06, *P* = .30). For level of depression, there was no significant difference between the MIDonline intervention and the control group at preassessment (F_1,201_ = 0.56, *P* = .45), postassessment (F_1,178_ = 0.03, *P* = .86), or at the follow-up assessment, (*F*_1,175_ = 1.71, *P* = .19). Within group effect sizes for depression literacy were −1.78 (MIDonline) and −0.07 (control); for personal stigma, they were 0.83 (MIDonline) and 0.06 (control); for perceived stigma, they were 0.14 (MIDonline) and 0.16 (control); and for depressive symptoms, they were 0.10 (MIDonline) and 0.10 (control).

**Conclusions:**

Current results suggested that the Internet may be a feasible and effective means for increasing depression knowledge and decreasing personal stigma in non–English-speaking immigrant populations residing in English-speaking countries. The lack of change in perceived stigma in this trial is consistent with results in other trials examining online depression stigma interventions in English-speaking groups.

**Trial Registration:**

ISRCTN76460837; http://www.controlled-trials.com/ISRCTN76460837 (Archived by WebCite at http://www.webcitation.org/5xjxva4Uq)

## Introduction

Low levels of depression literacy (also called depression knowledge) and high levels of stigma associated with mental disorders may be barriers to seeking help from health professionals [1-3]. Previous research has indicated that stigma associated with having a mental disorder is more prominent in non–English-speaking immigrant communities, especially among people born in Greece [4]. In 2006, Australia was home to over 110,000 Greek-born and 199,100 Italian-born people, with Melbourne having the largest Greek-born population outside of Greece [5]. In addition, the 2 most common of the 400 non-English languages spoken at home in Australia in 2006 were Italian and Greek, accounting for 1.6% and 1.3% respectively of the languages spoken at home among the Australian population [5]. However, despite non–English-speaking people comprising a substantial proportion of Australia’s population, with Italian-born and Greek-born making up the third and sixth largest non–English-speaking overseas-born groups living in Australia [5], there is a lack of research into depression literacy and stigma related to depression in these groups.

Online psychoeducational interventions have been reported to be effective in increasing depression literacy and reducing personal stigma (stigma toward people with a mental illness) related to depression in English-speaking populations [6]. Online information and psychological interventions have also been found to be effective in the treatment of depressive symptoms in adults (eg, [7-9]) and in addressing stigma related to mental health [10]. However, there is a lack of culturally appropriate evidence-based educational interventions for depression for non–English-speaking immigrant communities especially in the Australian context. There are good reasons to target such interventions to middle- and older-aged immigrants of a non–English-speaking background, especially those who are Greek- and Italian-born. Compared with Anglo-Australians, people in these groups have been found to hold stronger stigmatizing attitudes toward mental illness such as schizophrenia and depression, they tend to be at higher risk of developing depression, and they have been found to underutilize professional psychological services [4,11]. In this study, we evaluated the impact of a new, open access, Web-based multilingual informational intervention, Multicultural Information on Depression online (MIDonline), on depression literacy, personal (personal stigma toward people with a mental illness) and perceived stigma (participants’ views about the probable attitude of the general community toward people with a mental illness), and depressive symptoms among Greek-born and Italian-born immigrants living in Melbourne, Australia.

## Methods

### Trial Design

This was a single centre, cross-sectional, parallel group, randomized controlled trial with balanced randomization (ie, 1:1), and with stratification at level of country. The study was conducted at Monash University, Melbourne, Australia.

### Participants

Participants were a community sample of 129 Greek-born and 73 Italian-born immigrants living in Melbourne, Australia. They were recruited between November 11, 2006, and June 6, 2009, by advertising the research project in Greek and Italian social and welfare clubs and in the print and radio media directed at Greek- and Italian-speaking residents of Melbourne. Eligibility criteria for the study included being 45 years of age or over and having been born in Greece or Italy. The total sample comprised 144 women and 58 men. The mean participant age was 65.4 years (SD 8.57, range 48 to 88 years). Monash University Committee on Ethics in Research Involving Humans granted approval for the study.

### Interventions

#### MIDonline Intervention

The intervention comprised the consumer section of the MIDonline website [12], which provides online multilingual information about depression designed for middle- to older-aged consumers from a non–English-speaking background. The material is available in Greek, Italian, and English. The website content incorporates information about symptoms and case studies of depression, how depression is diagnosed, related disorders, causes, treatment options, how to find a bilingual mental health professional and professional psychological care, stigma related to mental illness, and multilingual translated resources. The information is provided in a culturally relevant way. For example, case studies are representative of middle- to older-aged people of both genders who are Greek- and Italian-born.

**Figure 1:**
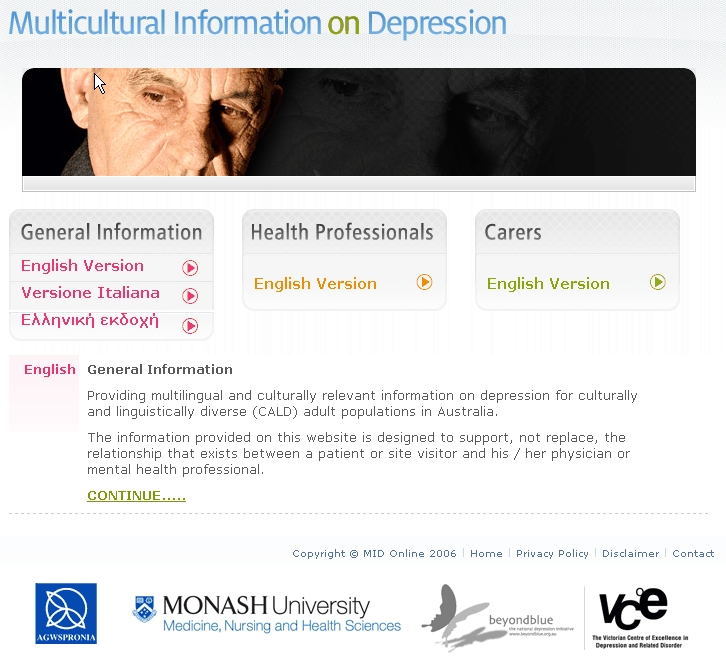
Screenshot of the MIDonline website intervention in English

**Figure 2:**
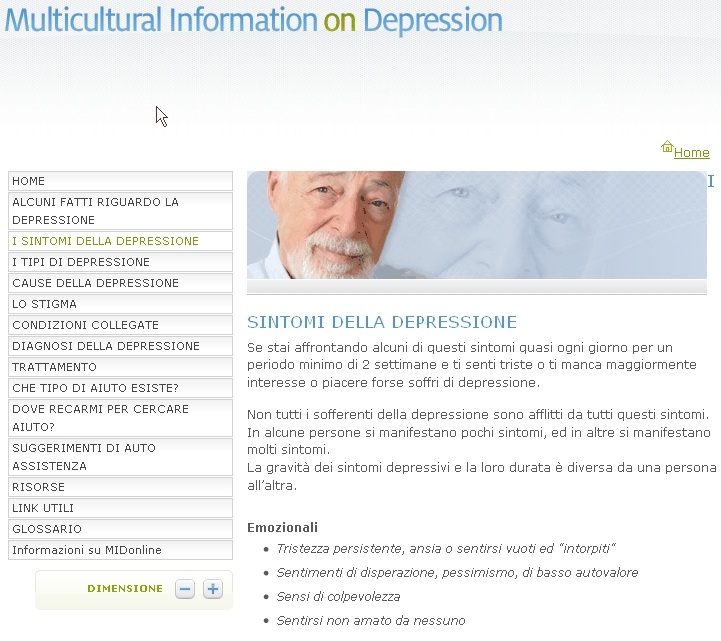
Screenshot of the MIDonline website in Italian

**Figure 3:**
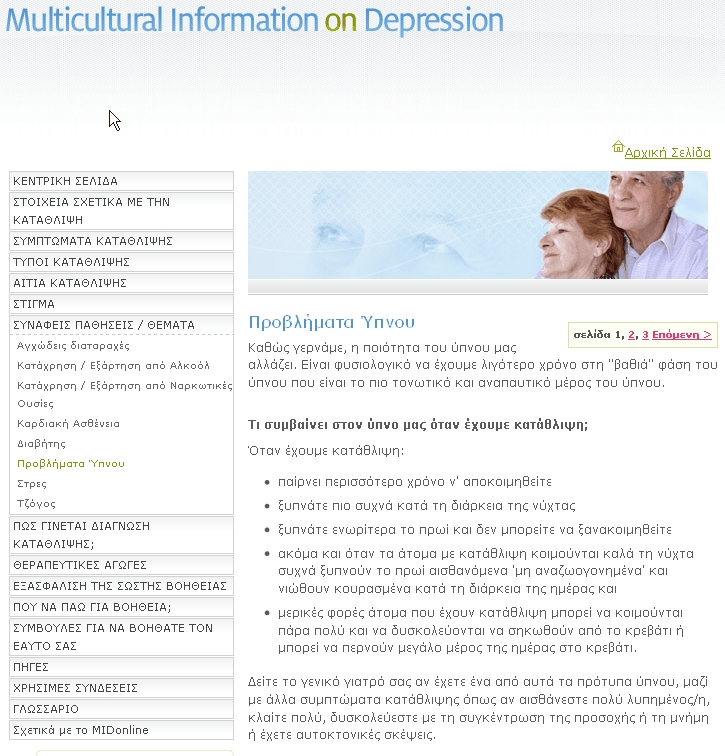
Screenshot of the MIDonline website in Greek

#### Control Condition

The control intervention consisted of a semistructured interview with a bilingual interviewer who asked open-ended questions relating to the participant’s beliefs about depression including the causes, symptoms, course and development, treatments, and outcomes of depression.

### Procedures

Greek-born and Italian-born participants who opted to take part in the research contacted the researchers who were listed in the advertisements related to the study. The interviewers organized a suitable time for eligible participants to take part either in the MIDonline intervention or the depression interview. Each participant was assigned a bilingual interviewer who administered the preassessments, postassessments, follow-up assessments, and interventions. The investigators were not involved in conducting any of the interviews or interventions.

All participants took part in two face-to-face sessions. During the first session, participants were administered a face-to-face preintervention questionnaire followed by the MIDonline intervention or the depression control interview and then a face-to-face postintervention questionnaire. The second session included a follow-up face-to-face questionnaire administered 1 week later. Questionnaires were completed in an interview format with the bilingual researchers due to the suspected low literacy levels among participants. All participants who took part in the study completed the preintervention, postintervention, and 1-week follow-up questionnaires. The follow-up questionnaire was completed an average of 7.95 days (SD 2.34) after participants had completed the postintervention questionnaire. The project was conducted from November 11, 2006, through June 6, 2009. The intervention delivery and data collection took place in a consultation room located at Monash University.

Prior to the study, all bilingual interviewers attended a training session during which they were provided with instruction and written verbatim protocols for conducting both the MIDonline intervention and the depression interview control condition.

For the MIDonline intervention, the participant and interviewer sat together in front of a computer displaying the MIDonline website. In the first 10 minutes of this session, interviewers explained that the purpose of the website was to increase knowledge about depression and instructed participants on how to navigate through the main sections of the website. These sections included information on depressive symptoms, case studies, how depression is diagnosed, treatment options, and stigma related to mental illness. Participants were then given an hour to read through the online material by themselves. Although the interviewers remained in the room during this period, they were instructed not to discuss the material with the participants. Participants only had access to the MIDonline website during the intervention on this one occasion.

Participants in the control condition took part in an interview in which interviewers asked participants a set of open-ended questions relating to the participant’s beliefs about the causes, important symptoms, course and development, treatments, and outcomes of depression. Both the MIDonline intervention and the control interview took 1 to 1.5 hours each to complete.

In all, six bilingual interviewers assisted participants through the MIDonline intervention or the depression attention control interview, four of whom spoke Italian and two of whom spoke Greek. All interviewers had experience conducting bilingual interviews and were registered psychologists (except for one Greek bilingual interviewer and one Italian bilingual interviewer, each of whom was a probationary psychologist). Both the intervention and the control interview were undertaken in the participant’s language of choice (either Greek or Italian). All participants provided written informed consent prior to participating in the study.

**Figure 4:**
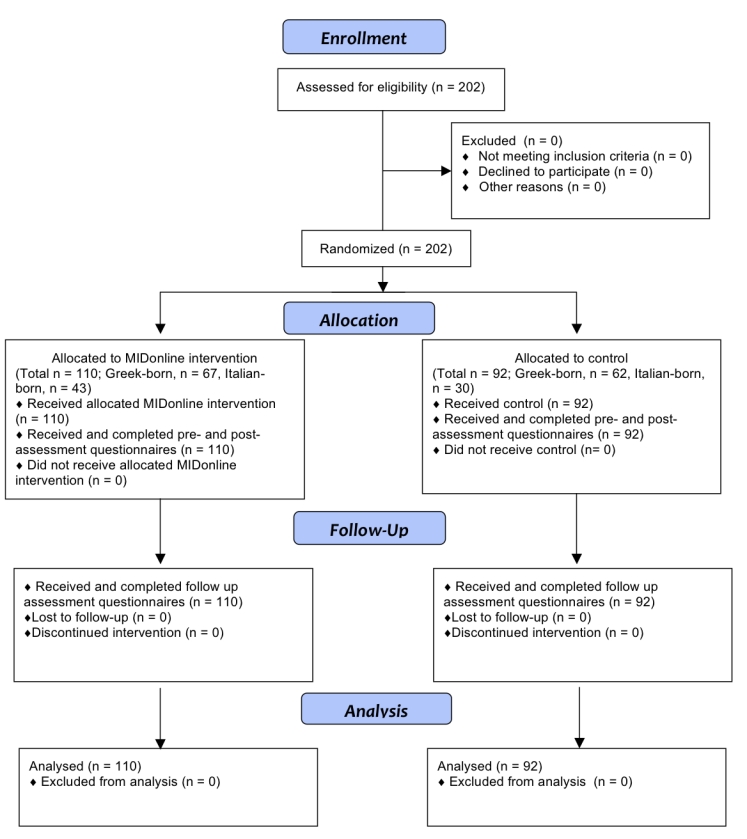
Flow of participants through research trial

### Participation

Figure 4 displays the participant flowchart for the trial according to consolidated standards of reporting trials (CONSORT) 2010 guidelines [13].

### Measures

The face-to-face questionnaires incorporated measures of sociodemographic status, personal and perceived stigma, depression severity, and clinical history. All self-report scales were translated from English into Greek and Italian for the purpose of this study by the first author (LK) and other bilingual psychologists. All item translations were reconsidered by a second bilingual psychologist and researcher. More difficult or ambiguous items were examined for meaning with lay members of the Greek and Italian communities and other mental health professionals working with these communities at the time. This method was preferred over sequential/back translation methods by an interpreter as it capitalized on the expertise of bilingual mental health professionals [4]. Validity was checked by examining the psychometric properties of the scales (factor structure and internal consistency) after data were collected and preceding any further analysis.

#### Sociodemographic Variables and Clinical History

The following sociodemographic variables were measured: age in years, gender, marital status (married, widowed, separated, single), birthplace and currently working (yes/no), and length of stay in Melbourne (in years). Level of education was measured on a 7-point scale (did not attend school, did not complete primary, completed primary, completed some secondary school, completed all secondary school, completed some tertiary, completed tertiary). Occupation level was measured on an 8-point scale (never did paid work, unskilled/semiskilled work, qualified tradesperson, clerical/office work, manager of a small business/shop/farm, manager of a small organization/company, professional requiring some university education, professional requiring high level of university education). Current living situation was measured with a 5-point scale where participants were asked about whether spouse, children, siblings, in-laws, parents, or other relatives were living at home with them. The Greek or Italian identity respectively with responses measured on a 4-item scale (not at all, a little, much, very much) with higher scores indicating greater ethnic identification. Items examined how Greek or Italian the participant felt, how important being Greek or Italian was to them, and to what extent they maintained the Greek or Italian way of doing things. Using the same rating scales, all participants were asked to rate the extent to which they considered themselves as having an Australian identity, how Australian the person felt, how important being Australian was to them, and to what extent they maintained the Australian way of doing things. English language proficiency was measured with a 5-item scale with responses measured on a 5-point scale (poor, fair, good, very good and excellent). Items depicted situations of different communicative difficulty and included shopping, regular banking, attending an English-speaking doctor, discussing important finances with the bank manager, and writing on formal business. Memory functioning was measured with a 6-item scale that asked participants about their memory of people’s names, stories, appointments, dates, news, and grocery lists. Responses were measured on a 5-point scale (much poorer than average, a little poorer than average, average, a little better than average, much better than average). Higher scores indicated better memory functioning. Participants were asked whether they were currently receiving treatment for an emotional or psychological problem (yes/no) and what type of treatment was being received and by whom. Level of depressive symptoms was measured with the Beck Depression Inventory–II [14].

#### Outcome Measures

The primary outcome measures were levels of depression literacy, personal and perceived depression-related stigma, and severity of depressive symptoms.

#### Depression Literacy

Depression literacy was assessed using translated and adapted versions of the D-Lit scale [6] which is a 22-item true/false test of knowledge about depression. A higher score on this scale indicated greater literacy. An example of an item on the D-lit is: “People with depression may feel guilty when they are not at fault*.”* Of the original items, 4 were replaced with the following items to better reflect the content of the MIDonline website. These items included: “Major depression is one of the leading causes of disability and loss of quality of life in the world,” “Causes of depression range from genetics, our environment, personality, our biochemistry and having other medical conditions,” “Nearly twice as many women as men are affected by depression,” and “Both antidepressants and talk therapies like CBT can be effective in treating depression.” The internal reliability analyses in the present sample indicated an alpha coefficient of .88 for the Greek version and .92 for the Italian version.

#### Depression Stigma

Changes in personal (reflecting participants’ personal attitudes) and perceived (reflecting participants’ beliefs about the attitudes of others) depression stigma were assessed using the 18-item Depression Stigma Scale [6]. Half of the items required participants to rate how strongly they personally agreed with a statement about depression (eg, “people with depression are unpredictable”). The other half of the items asked the participant to indicate what they thought most other people believed about the same issue (eg, “most people believe that people with depression are unpredictable”). Ratings were made on a 5-point Likert scale. Scores on the total scale range from 0 to 36 for the full scale and 0 to 18 for each of the two 9-item subscales, with higher scores indicating greater stigma. Cronbach alpha values for the total, personal, and perceived stigma scales were .68, .62, and .82 respectively for the Greek version. The correlation between the scores on the personal and perceived stigma scales was .13 (n = 129, *P* > .05). For the Italian version, Cronbach alpha values for the total, personal, and perceived stigma scales were .80, .76 and .72 respectively, and the correlation between the scores on the personal and perceived stigma scales was .39 (n = 73, *P* = .01).

#### Depressive Symptoms

Depression severity was measured with the Beck Depression Inventory–II (BDI–II) [14], which is a 21-item instrument for measuring severity of depression in adults. Responses to items covered the “past two weeks, including today.” Responses on the BDI–II items range from 0 to 6 with higher values indicating higher severity. The internal reliability analyses in the present sample indicated an alpha coefficient of .90 for the Greek version and .89 for the Italian version.

### Randomization

Participants were randomly assigned by the first author following a simple randomization procedure using a computerized list of random numbers to one of two intervention groups (either the MIDonline intervention (n = 110) or the control group (n = 92) using a 1:1 allocation with stratification at level of country). The sequence of numbers was concealed until the intervention was assigned. Interviewers and participants were not blinded to condition assignment. Participants contacted the bilingual interviewers who were listed on an advertisement to take part in the study.

### Sample Size

To detect a significant difference in depression literacy of at least 2 points (based on data reported by Griffiths et al [6]) with a two-sided 5% significance level and a power of 80%, a total sample of 128 (64 participants in each group) was required.

### Statistical Analyses

Differences between sociodemographic variables were examined using chi-square analyses. SPSS version 17 (IBM Corporation, Somers, NY, USA) was used to analyze the data.

To examine differences between intervention groups at baseline, individual analyses of variance (ANOVAs) were performed with preintervention depression literacy, personal stigma, perceived stigma, and level of depression scores.

To examine group differences at postintervention, individual analyses of covariance (ANCOVAs) were performed on the postintervention depression literacy, personal stigma, perceived stigma, and level of depression scores. The sociodemographic variables that differed between groups at baseline were used as covariates in all ANCOVA analyses. Covariates for these analyses included Australian identification, currently living alone, living arrangements, speaking English with an English-speaking doctor, and memory functioning. All covariates were dichotomized prior to use in the ANCOVA analyses. Preassessment depression literacy, personal stigma, perceived stigma, and level of depression were also used as covariates in the individual ANCOVA postassessment analyses.

To examine group differences at follow-up intervention, two sets of individual ANCOVAs were performed on follow-up intervention depression literacy, personal stigma, perceived stigma, and level of depression scores. Covariates for these analyses included all the demographic variables that differed between intervention groups at baseline. These included Australian identification, currently living alone, living arrangements, visiting an English-speaking doctor, and memory functioning. The first set of individual ANCOVAs was performed using the demographic variables that differed at baseline and the preintervention score of the variable as covariates. The second set of individual ANCOVAs was performed using the demographic variables that differed at baseline and the postintervention score of the variable as covariates.

Effect sizes from pre to post, were calculated using Cohen’s d (standardized mean difference at immediate post) [15].

## Results

### Sample Characteristics

Table 1 summarizes the characteristics of the two birthplace groups in each condition. Overall, there were no statistically significant differences between the MIDonline and control groups on the characteristics measured. The mean age for the total sample was 65.4 years (SD 8.57). There was no significant difference in participant age for the two conditions: MIDonline mean age 65.6 years (SD 8.1), control mean age 65.2 years (SD 9.0) (F_1, 201_ = 0.10, *P* > .05, n = 202). The percentages of women allocated to each condition did not differ significantly (60 out of 110 [54.5%] participants were allocated to MIDonline, and 42 out of 92 [45.5%] participants were allocated to the control condition, χ^2^_1_, = 0.57, *P* > .05, n = 202).

There was no significant difference in the level of education across the conditions (χ^2^_6_ = 11.01, *P* > .05), with the majority of participants reporting that they had completed all of primary school or some secondary school. The majority of Greek-born and Italian-born participants in the MIDonline and control conditions were married, living with their spouse, had been mainly unskilled or semiskilled workers, and were currently retired. Overall, very few participants were recent arrivals to Australia, with the majority having lived in Melbourne for an average of 43.8 years (SD 9.0). There were no differences found for length of stay for the two conditions; the mean for participants in the MIDonline group was 43.9 years (SD 9.89), and the mean for participants in the control group was 43.6 years (SD 8.34) (F_1, 201_ = 0.06, *P* > .05, *n* = 201).

The majority of the sample rated their English proficiency as “good” for very simple situations like shopping (χ^2^_3_ = 5.14, *P* = .16, n = 202), but “poor/fair” for more difficult situations such as doing their regular banking (χ^2^_3_ = 3.50, *P* = .32, n = 202), visiting an English-speaking doctor (χ^2^_3_ = 7.76, *P* = .05, n = 202), discussing their finances (χ^2^_3_ = 2.51, *P* = .47, n = 202), and important business (χ^2^_3_ = 5.55, *P* = .13, n = 202). The majority of the sample reported having “average” memory functioning. In addition, the majority of the sample was not receiving treatment for a psychological or emotional problem. However, those who were receiving treatment were doing so from a general practitioner, psychologist, or psychiatrist, and treatment included taking medication.

For the Greek-born, there was no significant difference in the endorsement of Greek or Australian identification across conditions. Overall, the majority of Greek-born participants identified “very much” with being Greek and identified “much” with being Australian. However, for the Italian-born, there was a significant difference in the endorsement of Italian and Australian identification across conditions. Whereas the majority of Italian-born participants in the MIDonline condition identified “much” with being Italian and “a little” with being Australian, the majority in the control condition identified “very much” with being Italian and “very much” with being Australian.

**Table 1:** Demographic characteristics of respondents for the MIDonline and control intervention conditions

		MIDonline	Control	
		(n = 110)	(n = 92)	
Variables		n (%)	n (%)	*P* Value
**Gender**				.45
	Male	34 (30.9%)	24 (26.1%)	
	Female	76 (69.1%)	68 (73.9%)	
**Married**				.21
	Yes	35 (31.8%)	22 (23.9%)	
	No	75 (68.2%)	70 (76.1%)	
**Greek identification**				.24
	Not at all/a little	6 (9.0%)	7 (11.9%)	
	Much	17 (25.4%)	22 (37.3%)	
	Very much	44 (65.7%)	30 (50.8%)	
**Italian identification**				.07
	Not at all/a little	7 (16.3%)	8 (26.7%)	
	Much	23 (53.5%)	8 (26.7%)	
	Very much	13 (30.2%)	14 (46.7%)	
**Australian identification**				.03
	Not at all/a little	52 (47.3%)	31 (34.8%)	
	Much	45 (40.9%)	35 (39.3%)	
	Very much	13 (11.8%)	23 (25.8%)	
**Currently living alone**				.01
	Yes	22 (20%)	7 (7.6%)	
	No	88 (80%)	85 (92.4%)	
**Living arrangements**				.03
	with spouse	23 (22.5%)	7 (8.4%)	
	with children	55 (53.9%)	50 (60.2%)	
	with other relatives	24 (23.5%)	26 (31.3%)	
**Level of education**				.14
	No/incomplete primary	17 (15.5%)	14 (15.2%)	
	Completed primary	39 (35.5%)	46 (50%)	
	Some secondary school	30 (27.3%)	19 (20.7%)	
	All secondary school	15 (13.6%)	5 (5.4%)	
	Some/completed tertiary	9 (8.2%)	8 (8.7%)	
**Occupation**				.60
	Never worked	4 (3.7%)	6 (6.6%)	
	Unskilled	65 (60.7%)	52 (57.1%)	
	Tradesperson/clerical	35 (32.7%)	28 (30.8%)	
	Manager/professional	3 (2.8%)	5 (5.5%)	
**Working now**				.11
	Yes	26 (23.9%)	31 (34.1%)	
	No	83 (76.1%)	60 (65.9%)	
**Memory Functioning Index**				<.001
	Above average	6 (5.5%)	24 (26.1%)	
	Average	103 (94.5%)	68 (73.9%)	
**Receiving psychological treatment**				.26
	Yes	9 (8.2%)	12 (13%)	
	No	101 (94.5%)	80 (87%)	
**Treatment from whom?**				.24
	No one	101 (91.8%)	81 (88%)	
	Psychologist	7 (6.4%)	5 (5.4%)	
	GP	2 (1.8%)	3 (3.3%)	
	Psychiatrist	0 (0%)	3 (3.3%)	
**What type of treatment?**				.08
	None	101 (91.8%)	81 (88%)	
	Counseling	3 (2.7%)	0 (0%)	
	Medication	6 (5.5%)	11 (12%)	

### Test-Retest Reliability of the Translated Scales

The test-retest reliability measures (Pearson’s correlation coefficients) based on pretest and follow-up test data for the combined control and MIDonline conditions for the Greek translations of all scales were .91 (n = 128) for the depression literacy scale, .80 (n = 128) for the personal stigma subscale, and .83 (n = 128) for the perceived stigma subscale (*P* < .001 in each case). For the Italian translations of all scales the test-retest reliability measures based on pretest and follow-up test data for the combined control and MIDonline conditions were .88 (n = 71) for the depression literacy scale, .78 (n = 71) for the personal stigma subscale, and .65 (n = 71) for the perceived stigma subscale (*P* < .001 in each case).

**Table 2:** Mean depression literacy, personal and perceived stigma and level of depression scores for each intervention group over time and associated *P* values

		MIDonline	Control	
		(n = 110)	(n = 92)	
		Mean (SD)	Mean (SD)	*P* Value
**Depression literacy**				
	Preintervention	10.61 (3.28)	8.17 (4.29)	< .001
	Postintervention	17.43 (3.99)	8.03 (4.33)	< .001
	Follow-up	16.84 (3.58)	8.22 (4.33)	< .001^a^, .01^b^
**Personal stigma**				
	Preintervention	18.38 (4.78)	18.44 (4.66)	.92
	Postintervention	14.69 (3.64)	18.35 (4.57)	< .001
	Follow-up	15.02 (3.95)	17.67 (4.73)	.001^a^, .06^b^
**Perceived stigma**				
	Preintervention	22.61 (4.39)	20.76 (5.40)	.008
	Postintervention	21.65 (4.49)	21.06 (5.74)	.44
	Follow-up	21.95 (4.13)	20.29 (5.55)	.30^a^, .03^b^
**Level of depression**				
	Preintervention	8.10 (7.84)	8.94 (7.82)	.45
	Postintervention	7.26 (7.64)	8.13 (7.53)	.87
	Follow-up	6.34 (6.60)	8.26 (7.88)	.18^a^, .19^b^

^a^ ANCOVA employed the preintervention measure of the variable as a covariate.

^b^ ANCOVA employed the postintervention measure of the variable as a covariate.

### Effects for Depression Literacy

Table 2 shows the mean depression literacy scores by intervention group over time and associated *P* values of the ANCOVAs performed. The effect of the intervention on depression literacy was examined by conducting an ANCOVA on the postassessment depression literacy score adjusting for baseline depression literacy (as well as Australian identification, currently living alone, living arrangements, speaking English to an English-speaking doctor, and memory functioning). This analysis revealed that those in the MIDonline intervention group showed a substantial increase in depression literacy scores compared with those in the control group after controlling for baseline depression literacy scores and relevant demographic variables (F_1,178_ = 144.99, *P* < .001). A similar analysis was conducted on the follow-up intervention scores with the ANCOVA demonstrating superior depression literacy for the MIDonline intervention than the control group after controlling for baseline depression literacy and relevant demographic variables (F_1,178_ = 129.13, *P* < .001) (see Table 2).

Finally, an additional ANCOVA was conducted to examine whether there were any differences between the MIDonline and control groups in depression literacy at the follow-up intervention stage after adjusting for the postintervention depression literacy score and the demographic covariates that differed at baseline. This analysis indicated that there was a significant but small reduction in the intervention effect on depression literacy at follow-up (F_1,176_ = 6.35, *P* < .01).

### Effects for Personal Stigma

There was no significant difference between the MIDonline and control groups for baseline personal stigma (see Table 2). ANCOVAs on the postintervention and follow-up personal stigma scores (adjusting for the preassessment personal stigma score and the demographic variables that differed between intervention groups at baseline) showed that MIDonline was associated with lower postintervention (F_1,178_ = 38.75, *P* < .001) and follow-up (F_1,176_ = 11.08, *P* = .001) personal stigma scores than the control group. However, a further ANCOVA of the follow-up personal stigma measures controlling for postintervention personal stigma levels indicated that there was a trend toward a small reduction in the effect at follow-up (F_1,176_ = 3.65, *P* < .06) (see Table 2).

### Effects for Perceived Stigma

The baseline preassessment perceived stigma score was significantly higher for the MIDonline group than the control group (see Table 2). An ANCOVA on postassessment (F_1,178_ = .60, *P* = .44) and follow-up (F_1,176_ = 1.06, *P* = .30) perceived stigma yielded no significant differences between the intervention and control group on this measure. However, an ANCOVA on the follow-up perceived stigma assessment score adjusting for the postassessment perceived stigma score showed group differences with those in the MIDonline intervention showing a small increase in perceived stigma scores relative to control in the period between postintervention and follow-up (F_1,__176_ = 4.91, *P* < .03).

### Effects for Level of Depression

There was no significant difference between the MIDonline and control groups for baseline level of depression (see Table 2). ANCOVAs on the postintervention (F_1,178_ = .03, *P* = .86) and follow-up (F_(1,175)_ = 1.71, *P* = .19) personal stigma scores showed no differences on level of depression between groups.

### Effect Sizes

The pre-post Cohen’s d effect sizes for depression literacy were −1.78 (MIDonline) and −0.07 (control) respectively. The corresponding effect sizes for personal stigma were 0.83 (MIDonline) and 0.06 (control) and perceived stigma −0.14 (MIDonline) and 0.16 (control). The effect sizes for level of depression were 0.10 (MIDonline) and 0.10 (control).

## Discussion

This study demonstrated that there were significant differences between the intervention group and the control group for depression literacy and personal stigma scores but not for perceived stigma or level of depression scores.

The finding that an educational intervention can increase depression literacy and reduce personal stigma related to depression has been reported previously [6]. However, to our knowledge, this is the first study to demonstrate such benefits among non–English-speaking immigrants to an English-speaking country. Strategies in the MIDonline website that may have contributed to the stigma reduction effect include the reinforcement of the message that depression is treatable and details of effective treatments, the use of culturally appropriate case studies, listings of bilingual mental health professionals, and information about culturally appropriate self-help strategies.

The identification of an intervention that will decrease personal stigma in this group is important given that the baseline levels of personal stigma for the two immigrant groups were higher than have previously been documented for the general, predominantly Anglo-Australian, adult population in previous research [6] and that previous research has found that personal depression stigma is higher in ethnic populations than English-speaking populations [6,16,17].

The finding that the depression Internet intervention did not decrease perceived stigma levels in Greek-born and Italian-born immigrants is consistent with a previous Internet intervention study that reported similar outcomes for an English-speaking community sample with elevated depressive symptoms [6]. The MIDonline website emphasizes that emotions can be changed by changing thoughts and behaviors, which may have led participants to perceive that others believe that depression is controllable by the depressed person and, hence, the fault of the depressed person. In addition, MIDonline described the history and current widespread stigmatizing beliefs about mental illness among Greek-born and Italian-born communities. This information may have reinforced the belief that stigmatizing attitudes toward mental illness are still widely and strongly held in the wider Greek-born and Italian-born immigrant communities. These findings suggest that no single approach is likely to reduce all aspects of mental health–related stigma [17].

The finding that the change in perceived stigma between postintervention and follow-up differed for the MIDonline and control groups is more difficult to interpret. Inspection of the scores suggests that the effect was primarily due to a reduction in perceived stigma among the control participants. It is conceivable that contact with interviewers among the control group led to a delayed reduction in the perception of stigma among that group, whereas, as noted above, the MIDonline content reinforced participants’ perceptions of stigma in others. Alternatively, the finding may be a spurious result attributable to statistical error associated with multiple comparisons. Further research is needed to investigate this finding.

Low literacy has previously been shown to be a barrier to finding health information for adults from an ethnic background, particularly on the Internet and independent of Internet and computer literacy [18]. Many people from a non–English-speaking ethnic background worldwide lack access to mental health–related information that meets their linguistic, cultural, or literacy needs [19]. The current study is a step toward addressing these issues by matching text readability more closely to the literacy levels of our target audience by using language targeted to school grade level 7 or 8.

Viewing the MIDonline website did not result in any changes to level of depression. This contrasts with findings from a systematic review that found that psychoeducational interventions were associated with reduced depressive symptoms relative to control [20]. There are two possible explanations for this discrepancy. First, participants in the current study were not selected on the basis of depressive symptomatology or depression, and the average baseline symptom scores fell well below the BDI-II cutoff score of 14 for mild depression. More significantly, however, the 2-week time frame used in the BDI-II for assessing presence of symptoms was not appropriate for immediate and 1-week follow-up time frames. Further research is required to determine if informational interventions can reduce depressive symptoms among those with elevated depressive symptoms in this population.

### Limitations

The study has some limitations. The samples may not have been representative of the broader Greek-born and Italian-born ethnic communities. Both samples were obtained by advertising the study in ethno-specific welfare and social groups and included individuals who volunteered to participate and who were willing to undertake an Internet intervention to increase their knowledge of depression. The fact that this methodology excluded individuals who do not frequent such clubs and organizations may limit the generalizability of the findings.

As part of this study, instrumentation was translated and, where appropriate, modified for the purpose of examining depression literacy and stigma in Greek-born and Italian-born people. The translated instruments may have been measuring different concepts than those measured by the English version of the scale.

Previous studies have found that socially desirable responding influences symptom reporting in non–English-speaking populations [21-23]. Such effects may occur for stigma measures. Future work is required to evaluate the effect of social desirability on the findings. Another limitation of the current study is that the same interviewer was used for the assessment and intervention phases of the study providing a potential source of bias. Similarly, the involvement of the first author in the randomization process had the potential to introduce allocation bias.

A final limitation of the study is that the posttesting and follow-up testing occurred soon after completion of the intervention. Longer follow-up periods are required to examine the sustainability of effects. In addition, an interviewer was present during the MIDonline intervention to assist participants in the navigation of the consumer section of the website. Hence, the current intervention involved some, albeit minimal, face-to-face interviewer guidance, and it is not known if automated distal delivery will yield similar findings in this population.

### Future Research

The current study demonstrated the effectiveness of a culturally appropriate depression information intervention in increasing depression literacy and reducing personal stigma among Greek and Italian immigrant populations, who have been shown previously to hold strongly stigmatizing attitudes toward mental illness. Future work is required to examine whether such interventions are effective in reducing stigma among other non–English-speaking immigrant populations and in those with a current or past history of depression or their family members. Future studies should also investigate the information technology literacy and health-related Internet use among the older, non–English-speaking population.

In the current study, the magnitude of effect sizes for the Web-based intervention are very large, and in a public health context, these effect sizes could translate into large gains for large numbers of people at a low cost [24]. The intervention warrants further investigation as a method for delivering depression literacy and stigma reduction programs for depression in other non–English immigrant groups, particularly in an automated format without guidance.

## Abbreviations

ANOCOVA: analysis of covariance
ANOVA: analysis of variance
BDI–II: Beck Depression Inventory–II
CONSORT: consolidated standards of reporting trials
MIDonline: Multicultural Information on Depression online
